# Enhancing Trauma Care in Tertiary Hospitals: Addressing Gaps and Pathways to Improvement

**DOI:** 10.1155/emmi/2780171

**Published:** 2025-02-17

**Authors:** Eesha Yaqoob, Shahzad Ali Khan, Dua Abbas Zaidi, Bipin Chaurasia, Fazal Ullah Khan, Kyriacos Evangelou, Nimirta Sahitia, Saad Javed

**Affiliations:** ^1^Department of Public Health, Violence, Injury Prevention and Disability Unit, Health Services Academy, Ministry of National Health Services Regulations and Coordination, Government of Pakistan, Islamabad, Pakistan; ^2^Department of Public Health, Violence, Injury Prevention and Disability Unit, Health Services Academy, Islamabad, Pakistan; ^3^Department of Neurosurgery, Neurosurgery Clinic, Birgunj, Nepal; ^4^Department of Neurosurgery, Holy Family Hospital, Rawalpindi Medical University, Rawalpindi, Pakistan; ^5^Department of Medicine, National and Kapodistrian University of Athens, Athens, Greece; ^6^Department of Neurosurgery, Brain Surgery Hospital, Violence, Injury Prevention and Disability Unit, Health Services Academy, Ministry of National Health Services, Regulations and Coordination, Islamabad, Pakistan

**Keywords:** emergency services, healthcare infrastructure, Islamabad, Rawalpindi, trauma care

## Abstract

**Background:** Trauma is a major cause of morbidity and mortality globally, with road traffic accidents projected to be the leading cause of death by 2030. In developing countries like Pakistan, trauma patients face significant challenges in receiving timely and effective care. This study aimed to evaluate trauma centers in tertiary care hospitals in the twin cities of Pakistan to highlight gaps and pitfalls in trauma patient management.

**Methods:** A descriptive cross-sectional study was conducted using the World Health Organization's Hospital Emergency Unit Assessment Tool (HEAT) at five major public sector hospitals in Islamabad and Rawalpindi. Data collection involved collaboration between the Violence, Injury Prevention and Disability Unit and key informants, including Emergency Room in-charges and Heads of Departments. Information on trauma protocols and guidelines was gathered.

**Results:** All hospitals provided 24/7 emergency services with access to operating rooms and laboratories. However, significant disparities were found in equipment availability, particularly portable X-rays (40% availability) and RDT/HIV testing (20% availability). Protocol adherence varied, with 80% of hospitals having clinical management protocols but only 20% having specific protocols for conditions like asthma exacerbation and maternal hemorrhage. This study identifies infrastructural deficiencies and highlights systemic barriers that contribute to inadequate trauma care delivery, underscoring the need for targeted reforms.

**Conclusion:** The study highlights significant gaps in trauma care management in Pakistani tertiary care hospitals, including shortages of personnel, infrastructure deficiencies, and lack of standardized protocols. These findings underscore the urgent need for systemic improvements in trauma care delivery. Recommendations include increased investment in medical infrastructure, addressing staffing and training deficiencies, and standardizing clinical management protocols to enhance trauma care outcomes and reduce morbidity and mortality rates in Pakistan. This research contributes novel insights into the specific barriers faced by trauma care systems in Pakistan, which have not been previously addressed in existing literature.

## 1. Introduction

Trauma is a major cause of morbidity and mortality across the globe, including in both developed and developing countries [1]. Road traffic accidents are one of the major causes with which patients present to emergency (ER) units. According to the second Global Status Report on Road Safety, road traffic accidents will become the leading cause of death globally due to polytrauma by 2030 [2]. In developing countries like Pakistan, occupational injuries also contribute significantly to the number of trauma patients presenting to tertiary care hospitals. Most of these injuries can be attributed to unregulated industries [3]. Despite previous studies identifying downward trends in trauma care efficacy, this study aims to provide a comprehensive analysis of specific gaps within Pakistani tertiary hospitals' trauma care systems that have not been thoroughly explored yet.

The concept of the golden hour in trauma management, coined in the 1970s, states that critically injured patients receiving definitive care within the first 60 minutes of the incident have the best prognostic indices [4]. This approach is globally appreciated and thought to be clinically plausible. However, a number of studies in the literature have argued against this approach. Recent data indicates that increased total prehospital care time does not necessarily increase mortality; rather, the level of care provided upon hospital arrival is associated with decreased mortality [5]. A recent study in Sweden states that care provided at hospitals with dedicated trauma centers is associated with up to a 40% decline in 30-day mortality [6]. Multiple studies show that treating severely ill trauma patients in hospitals with fully equipped trauma centers is associated with a reduced mortality rate of 15%–32% [7].

Pakistan, being the sixth most populous country in the world, has a substantial burden of trauma patients presenting to resource-limited hospitals [8]. National surveys depict trauma as a major cause of morbidity and mortality, particularly among young males [8]. Over the last decade, multiple studies have highlighted the burden of trauma patients and the pitfalls in their management. Farooq et al. have depicted significant quality gaps in the prehospital and in-hospital care of patients with polytrauma resulting from road traffic accidents [9].

The World Health Organization (WHO) has established guidelines for the effective management of trauma patients. These guidelines recognize the disparity in outcomes for trauma patients between low-income and high-income countries. Mortality resulting from trauma is around 60% in middle-income countries compared to 30% in high-income countries [10]. This disparity is also observed at rural and urban levels. A study conducted to highlight the in-hospital disparity in mortality among trauma patients in rural and urban centers states that mortality rose from 6% in urban centers to 36% in rural centers [11].

The pitfalls in the management of trauma patients in low-income and middle-income countries primarily include human resources and physical resources [12]. According to the Pakistan Medical and Dental Council, Pakistan has about 0.2 million registered medical practitioners across all specialties, including general practitioners who have scarce knowledge and training regarding trauma management. Furthermore, little importance is given to the training of available staff. This is a strong contributing factor to the comparatively high mortality among trauma patients in developing countries like Pakistan [12].

The availability of physical resources, including infrastructure, surgical equipment, and supplies essential for managing life-threatening injuries, is pivotal to effective trauma management [12]. There is a lack of inexpensive surgical equipment, such as chest tubes, in many centers across Pakistan [13]. Tertiary care hospitals are better equipped compared to secondary centers for managing trauma. However, these tertiary care hospitals also have a long way to go to meet the standards of international trauma centers [13].

This study aims to evaluate the trauma centers of tertiary care hospitals in the twin cities of Pakistan. With this study, we aim to highlight important gaps and pitfalls in the management of trauma patients, chiefly their management in the trauma centers of ER departments after arrival at the hospital.

## 2. Materials and Methods

This study employs a quantitative research methodology with a descriptive cross-sectional study design, to assess the trauma care system in Pakistan comprehensively. A targeted sampling strategy was employed to ensure representation from the metropolitan twin cities of Rawalpindi and Islamabad, amongst the major urban cities in Pakistan with significant populations and healthcare infrastructure.

The study ensures representation from the major public sector hospitals within the twin cities, Islamabad, and Rawalpindi (Table 1). The selected healthcare facilities for this study encompass 1. Pakistan Institute of Medical Sciences (PIMS), 2. Federal General Hospital (FGH), 3. Polyclinic Hospital, 4. Holy Family Hospital, 5. Benazir Bhutto Hospital.

The WHO's Hospital Emergency Unit Assessment Tool (HEAT) was the data collection tool used for this assessment of the trauma care system. HEAT is a standardized tool that helps healthcare facilities assess their capacity to provide ER care. It also helps identify gaps in ER care delivery and targets interventions to strengthen it. The infrastructure of the institution, the availability of therapeutic services (including hours of operation), human resources, and clinical guidelines like checklists and protocols are all evaluated by the HEAT [14, 15].

The data collection involved a collaborative effort between the Violence, Injury Prevention, and Disability (VIPD) Unit, the Health Services Academy (HSA), and the Ministry of National Health Services, Regulations, and Coordination (MoNHR&C). Facilitation letters were issued to the hospitals to ensure cooperation, followed by meetings with the Administration of the hospitals. Key informants were identified to collect data and get the required information regarding trauma protocols and guidelines. These key informants included the Director of Medical Services (DMS), Medical Superintendent (MS), and ER in charge at the respective hospital. The collected data were analyzed through appropriate techniques with data entry facilitated through SPSS. Measures of central tendency, including means, were calculated to provide insights into key variables characterizing the trauma care system in Pakistan.

## 3. Results

All hospitals assessed were tertiary-level facilities providing ER services 24/7. However, significant disparities were found in equipment availability and protocol adherence. Notably:

Equipment Availability: Portable X-ray machines were available in only 40% of hospitals.

Protocol Adherence: While 80% had clinical management protocols, only 20% had protocols for critical conditions such as asthma exacerbation or maternal hemorrhage.

These findings reveal systemic barriers that contribute to ineffective trauma care delivery across these facilities.

The populations served by each facility varied significantly, with the FGH in Islamabad serving 400,000 people, the PIMS in Islamabad serving 737,662, the Polyclinic Hospital (PCH) in Islamabad serving 120,000, and both the Holy Family Hospital (HFH) and Benazir Bhutto Hospital (BBH) in Rawalpindi serving 5,500,000 each. Each hospital had designated rooms for the Emergency Room (ER).

ER visits per year also showed variation among the hospitals: 60,000 at FGH, 540,000 at PIMS, 28,800 at PCH, 367,171 at HFH, and 319,879 at BBH. Similarly, Outpatient Department (OPD) visits per year ranged from 288,000 at FGH to 1,281,078 at PIMS, with PCH, HFH, and BBH reporting 800,000, 658,716, and 548,762 visits, respectively. Inpatient Department (IPD) admissions per year were highest at PIMS (77,370) and lowest at FGH (3,800), with PCH (21,600), HFH (30,662), and BBH (26,983) in between. The ER bed count ranged from 50 at PCH to 126 at HFH, with PIMS, FGH, and BBH having 57, 60, and 100 beds respectively. Inpatient bed count was highest at PIMS (1,262) and lowest at FGH (60), with HFH, PCH, and BBH having 1,062, 900, and 800 beds, respectively. The number of operation theatres (OT) and annual ER operations varied as well, with FGH having 1 OT and 360 ER operations, PIMS 6 OTs and 44,345 ER operations, PCH 5 OTs and 28,800 ER operations, and both HFH and BBH having 3 OTs with 4781 and 4019 ER operations, respectively.

Essential equipment and infrastructure maintenance for the ER, such as clean running water, electricity, designated communication devices, paper-based and electronic unit charts, isolation rooms, designated waiting, resuscitation and triage areas, cardiac monitoring, PPE, oxygen, crash trolleys, and ambulances, were assessed. FGH and PCH reported somewhat availability of essential equipment and infrastructure maintenance, while PIMS, HFH, and BBH reported adequate availability. Four hospitals had 24/7 pharmacy and radiology services, whereas one provided these services for only 8 h a day. Wheelchairs, dedicated staff work areas, toilet facilities, handwashing facilities, and systems for stocking, managing, and dispensing in the ER were adequately available in one facility, somewhat available in three, and generally unavailable in one. Four hospitals had core-fixed providers permanently assigned to emergency units, consulting services with ER staff, and triage with category protocols, but only three applied systematic triages with syndromic surveillance. Neuro consulting services were unavailable in three hospitals, and psychiatry services were unavailable in two. ER access for social work, patient transport, and security for emergency personnel was generally adequate, except for social work services in one hospital and security in another.

All hospitals (Tables 2 and 3) supplied oxygen through a central pipe system, with oxygen stored in tanks and concentrators in four and three hospitals respectively. Essential diagnostic tests, including haemoglobin, FBC, PT, CTT, electrolytes, BUN, creatinine, lipase, cardiac markers, arterial blood gas (ABG) (except one hospital), CMBP testing, urine dipstick, urine pregnancy, and glucose testing, were performed in all facilities. However, only one hospital could run RDT and HIV testing. All hospitals could conduct ultrasounds, CT scans, and stationary X-rays, but only two provided portable X-rays.

Regarding clinical management protocols, four hospitals had them in place, but only one had specific protocols for asthma exacerbation, pneumonia, maternal hemorrhage, sepsis, and diabetic ketoacidosis. Transfer and safety protocols were available in three hospitals, while quality improvement protocols were generally inadequate, with moderate adequacy reported in two facilities. Most facilities recognized the importance of immediate interventions, with all of them adequately or somewhat adequately measuring and monitoring vital signs, performing airway, breathing, volume resuscitation, cardiac interventions, safe blood transfusion, and procedures for seizures, unconscious patients, and trauma care. However, one hospital reported no procedures for sepsis control, another reported inadequate essential equipment, and vaginal delivery, uterotonic drugs, and neonatal resuscitation were unavailable in two hospitals.

## 4. Discussion

The availability and differences in emergency services, equipment, and trauma protocols in tertiary hospitals in Pakistan are overviewed in our study. All hospitals were able to provide emergency treatment around the clock, with access to operating rooms and their labs. However, there are remarkable differences when it comes to equipment; portable X-ray machines were present in only 40% of hospitals, while just 20% could provide ABG services. RDT and HIV testing availability is also restricted, potentially hindering the diagnostic process. When it comes to protocol adherence, there are noticeable gaps in the adoption of standardized clinical management protocols, despite their establishment in most hospitals. Therefore, a unified approach to trauma care is crucial to ensure optimal patient-centered care.

Differences in service availability were marked, with complete pharmacy and radiology services for a limited time daily only provided by one hospital. On the contrary, 80% of institutions offer these services 24/7, highlighting a considerable disparity that undoubtedly impacts patient outcomes. Furthermore, comprehensive care provision is hampered by the scarce availability of essential diagnostic tools (i.e., portable X-rays, RDT and HIV testing, and ABG services), which represents an area in need of dramatic improvement.

According to the WHO, efficient trauma care systems encompass quick access to emergency services, availability of diagnostic equipment, and the employment of standardized treatment procedures to minimize death rates and ameliorate patient outcomes around the world [16]. Our study highlights multiple areas in which the trauma care facilities of Pakistan fall short in terms of global standards. The issue concerns not only the diagnostic tools but also the lack of defined care procedures, which set obstacles for healthcare practitioners and hinder the provision of trauma care that is both consistent and of high quality.

All these discrepancies between Pakistan and worldwide standards are a cause of worse patient outcomes, especially increased morbidity, and death rates [17]. The lack of necessary diagnostic tools and defined management methods leads to delays in patient treatment, which increase the number of complications and induce fatalities [18]. In underdeveloped countries, healthcare delivery faces even greater limitations: financial difficulties, restrictions in infrastructure, and unequal medical equipment availability further complicate comprehensive trauma care [19]. For these gaps to be addressed, investments in medical infrastructure, special training of healthcare practitioners, and the adoption of uniform trauma management guidelines nationwide are necessary. In closing disparities, international collaboration and assistance could play an important, adjuvant role. Lacking considerable change, public health in Pakistan will continue to suffer. To lower the burden of injuries and fatalities from motor vehicle accidents (the number one cause of death and disability worldwide), it is critical to improve trauma care systems [20].

The insistence on the availability of medical services and equipment stems from their importance for timely and successful triage and management of trauma patients as abstracted in the literature. Institutions that provide a complete range of treatments (both medical and surgical) can diagnose and treat trauma patients much more successfully, lowering complication and death rates significantly [21, 22]. Moreover, the faster vital services and equipment are made available, the better patient prognosis seems to be. Severely injured patients with rapid access to critical care units, blood transfusion services, and the operating room demonstrate lower mortality rates [23–25]. It is, therefore, crucial to guarantee resource availability to improve general trauma treatment standards in Pakistan and beyond.

The importance of having dedicated trauma centers is directly linked to improved patient outcomes observed following specialized care provision. Such institutions can pool resources and expertise to implement the best evidence-based care and practices that minimize mortality and complications postinjury [26, 27]. When trauma patients are initially assessed (i.e., triaged), it is imperative to take prompt medical action and swiftly transport them to suitable institutions as part of efficient prehospital care [28]. Research has highlighted how prehospital services (e.g., paramedic and ambulance) can improve survival rates, as they can ensure prompt attention and transfer to facilities equipped to handle their individual injury-related requirements and needs [29–31]. Especially in the first crucial hours postinjury, these components of trauma care become even more important. Early treatment initiation and advanced specialized trauma center capabilities can take advantage of this timing and integrate the discussed effective prehospital care with optimal results [32, 33].

To stratify patient treatment according to disease severity, formal, structured, and well-defined triage systems are necessary. The prioritization of patients who require immediate attention and thorough assessment of their medical requirements immediately upon arrival can remarkably improve their outcomes. When appropriately employed, triage systems have been shown to drastically lower fatality and waiting times for critically injured patients [34–37]. Additionally, committed, nonrotating ER doctors ameliorate service continuity and caliber, as these professionals have been specifically trained to make quicker and better choices. In contrast, hospitals with rotating staff frequently face inconsistencies in care provision, higher risks of malpractice, and more errors due to inexperience when dealing with ER department policies and procedures. Combining official triage methods with specialized, nonrotating ER healthcare providers can improve patient management, cut down on treatment times, and enhance patient satisfaction and outcomes [24].

Standardized protocols are necessary to ensure that all ER practitioners adhere to uniform evaluation and treatment standards. Consistency is the key to raising the overall effectiveness of trauma care, minimizing mistakes, and streamlining procedures [38]. Of course, ongoing training and education are the base to maintain the most recent and best practices. Continuous education and training programs for ER personnel uphold high standards of care and guarantee proficiency in the newest methods and tools [39]. Furthermore, ongoing education facilitates the continuous adoption of new procedures, recommendations, and guidelines. The adoption of established standards by trained ER physicians in specialized institutions is the cornerstone of improving trauma treatment effectiveness and caliber. Hospitals implementing such strategies report notable improvements in outcomes, with most importance placed on lower death rates and higher satisfaction [40, 41]. Therefore, it is imperative for Pakistan to invest in those areas for its healthcare to offer optimal trauma management.

At this point, it must be underscored that comprehensive trauma treatment heavily depends on consulting services across a wide range of disciplines, including -but not limited to-internal medicine, orthopedic surgery, anesthesiology, and neurosurgery. The specialized expertise of these services offers accurate assessment and diagnosis, guaranteeing that patients receive the best care possible tailored to their individual injuries. One of the major obstacles in the hospitals we included is the scarcity of consultation services. Patient management can be jeopardized if specialist consultation is initiated belatedly due to service unavailability. Extending beyond our primary point, one should not neglect that ER staff may be overworked and burnt out when forced to handle complicated situations falling outside their area of competence while anticipating specialist consultants to be reached [42]. In acute trauma settings, limited access to consultation services predisposes to suboptimal care, prolonged hospitalization, increased complications, and higher morbidity [43]. This situation emphasizes how important it is to prioritize and support consultation services in our country for thorough and prompt care.

For care in acute trauma settings to be standardized, however, a backbone of guidelines consisting of clinical management, transfer, and safety-associated components needs to be structured. Protocols like these minimize treatment approach variability (highly probable given the disparities observed across institutions in Pakistan) and improve the overall quality of care. According to the literature, the level and quality of clinical management and safety standards are integral to short- and long-term outcomes by directly influencing how prompt and effective interventions can be [44]. An example illustrating this is the considerable increase in survival rates and functional outcomes of patients with severe traumatic brain injury when the implementation of standardized guidelines ensures that no crucial treatment stages are missed [45]. Efficient transfer processes contribute to minimized delays in definitive care provision, even more critical for patients requiring urgent surgical intervention or specialized treatment unavailable at the initial point-of-care. Transfer protocols are, therefore, essential for patients to be promptly transferred to facilities equipped to provide the optimal level of care, customized to their injuries [46, 47].

Regarding the institutions we studied, infrastructure constraints such as antiquated buildings, insufficient space for patient management, and inadequate medical equipment seem to be a major issue. These restrictions can seriously impact the capacity to deliver prompt and efficient trauma care, prolong waiting times, and deteriorate outcomes. Another important problem is the lack of committed (i.e., nonrotating), qualified medical personnel, especially in ER and trauma care units. Insufficient specialist healthcare provider availability is known to induce a cascade of heavier workloads for current employees, elevated stress levels, lower care quality, and ultimately, worse patient outcomes [48]. Hospitals in Pakistan are also affected by significant resource constraints, the most important being the dearth of basic medical supplies and the restricted access to necessary equipment. These limitations ultimately hamper any crucial diagnostic procedures, efficient patient care delivery, and appropriate therapy administration [49].

Taking inspiration from effective methods in other countries, one possible way to overcome personnel and infrastructure issues would be to harness the power of telemedicine and remote consultation services [50]. This strategy can remove some of the strain on physical resources, facilitate access to care, and provide specialized medical expertise to impoverished regions [51]. It is also essential to invest in the education and training of the medical personnel assigned to trauma units to improve their skills and abilities. Furthermore, collaborating with medical facilities abroad for initiative exchange might provide fresh perspectives and knowledge. In this context, Pakistan can investigate public-private partnerships (PPPs) as a means of obtaining finance to modernize buildings, procure equipment, and bypass constraints. PPPs can prepare the ground for upgrades to hospital infrastructure, access to cutting-edge technology, and overall improvement in the standard of care, as demonstrated in other countries with success [52, 53].

To remedy the noted gaps, hospital managers and healthcare policymakers should prioritize the purchase of equipment and infrastructure such as portable X-ray machines and more specialized diagnostic tests. To adequately handle complex trauma situations, we propose for healthcare budgets to be raised, PPPs to be formed, and foreign assistance to be received. To address staffing and training deficits, it would be suggestable to implement strategies that facilitate ER professional recruitment, retention, and ongoing medical development. Policies should concentrate on establishing mandatory training programs, developing appealing career pathways for medical students interested in ER medicine and/or residents in training, and hiring committed, nonrotating ER healthcare providers. Procedures must be standardized, and hospitals should closely work with healthcare policymakers [44] to create and execute national trauma care standards based on worldwide best practices. A national trauma care committee should be formed to supervise the implementation of these protocols around Pakistan and function as the basic pillar that will evaluate the compliance of all institutions.

Future studies should investigate and assess how effectively policy measures can improve trauma management, with an emphasis placed on evaluating effects on patient outcomes and care delivery with enhanced infrastructure, staff availability, and standardized practices. Input from such research will provide the necessary empirical data that will guide policymakers and administrators to revise and change policies. Research on cutting-edge trauma care delivery that could work in underdeveloped countries like Pakistan is necessary [54] and includes community-based ER care models making use of available local resources, telemedicine, and mobile health technology. These studies can be a valuable source of information regarding low-cost scope-expanding and standard-of-care-raising methods in limited-resource environments, considering how much awareness of the socioeconomic factors contributing to trauma care in developing nations the establishment of therapeutic protocols requires [55]. Ultimately, the connection between the socioeconomic status and the frequency, intensity, and results of trauma care should be examined so policymakers can better address the underlying issues and put comprehensive prevention- and treatment-integrating public health strategies into practice.

## 5. Conclusion

The study highlights significant gaps in trauma care management within Pakistani tertiary hospitals, including shortages of personnel and infrastructure deficiencies. These findings provide novel insights into specific systemic barriers faced by healthcare providers, emphasizing an urgent need for systemic improvements in trauma care delivery.

Recommendations include increased investment in medical infrastructure, addressing staffing shortages through targeted training programs, and standardizing clinical management protocols tailored to local needs. By addressing these specific gaps identified through this research, stakeholders can significantly enhance trauma care outcomes and reduce morbidity and mortality rates among trauma patients in Pakistan.

## Figures and Tables

**Table 1:** Hospital information.

Sr. No.	Hospital name	City	Catchment population	Key informant's designation
H1	PIMS	Islamabad	737,662	Emergency room in-charge

H2	FGH	Islamabad	400,000	Emergency room in-charge

H3	Polyclinic hospital	Islamabad	120,000	Emergency room in-charge

H4	Holy family	Rawalpindi	5,500,000	Head of Department (HOD) of General Surgery and Dean of Postgraduate Education HOD/in-charge of Emergency HFH

H5	Benazir Bhutto Hospital	Rawalpindi	5,500,000	Head of Department (HOD) of General Surgery DMS

**Table 2:** Facility characteristics.

Indicators	Federal General Hospital (FGH)	Pakistan Institute of Medical Sciences (PIMS)	Polyclinic Hospital	Holy Family Hospital	Benazir Bhutto Hospital
Population served	400,000	737,662	120,000	5,500,000	5,500,000

Designated room for ER	Yes	Yes	Yes	Yes	Yes

Annual ER visits	60,000	540,000	28,800	367,171	319,879

Annual OPD visits	288,000	1,281,078	800,000	658,716	548,762

Annual IPD admissions	3800	77,370	21,600	30,662	26,983

ER beds	60	57	50	126	100

IPD beds	60	1262	900	1062	800

Number of OT	1	6	5	3	3

Number of ER operations	360	44,345	28,800	4781	4019

Opening hours ER	12 h	24 h	24 h	24 h	24 h
Opening hours lab	24 h				
Opening hours pharmacy	8 h				
Opening hours radiology	8 h				
Opening hours OT	24 h				

⁣^∗^Essential equipment and maintenance of infrastructure for ER available	Somewhat available	Adequate	Somewhat available	Adequate	Adequate

	Adequate	Somewhat available	Somewhat available	Generally unavailable	Somewhat available

⁣^∗^Clean running water, Electricity source, designated telephone/radio to communicate with other facilities, paper-based, electronic unit charts, isolation rooms for infectious diseases, designated waiting, resuscitation and triage area, cardiac monitoring in ER, PPE, oxygen in ER, crash trolley and ambulance for transfer patient.

**Table 3:** Equipment, diagnostic test and protocol availability in the facilities.

**Equipment, diagnostic test, and protocol availability in the facilities *N* (%)**			
	**Adequate**	**Some availability**	**Unavailable**

Equipment			
Oxygen in the emergency unit	2 (40%)	3 (60%)	0
Reporting system	4 (80%)	1 (20%)	0
Ultrasonography in the emergency unit	4 (80%)	1 (20%)	0
Stationary X-ray in the hospital	4 (80%)	1 (20%)	0
Portable X-ray in the hospital	2 (40%)	1 (20%)	3 (60%)
CT scan in the hospital	4 (80%)	1 (20%)	0
Diagnostic tests			
Arterial blood gas	4 (80%)	0	1 (20%)
Haemoglobin	5 (100%)	0	0
FBC	5 (100%)	0	0
PT	5 (100%)	0	0
CTT	5 (100%)	0	0
Electrolytes	5 (100%)	0	0
BUN	5 (100%)	0	0
Creatinine	5 (100%)	0	0
Lipase	5 (100%)	0	0
Cardiac markers	5 (100%)	0	0
CMBP	5 (100%)	0	0
Urine dipstick	5 (100%)	0	0
Urine pregnancy	5 (100%)	0	0
Glucose	5 (100%)	0	0
RDT and rapid HIV	1 (20%)	0	4 (80%)
Protocols			
Electronic system for registrations	3 (60%)	0	2 (40%)
Triage with category protocols	4 (80%)	0	1 (20%)
Systematic triage/syndromic surveillance	3 (60%)	1 (20%)	1 (20%)
Clinical management	4 (80%)	0	1 (20%)
AS-P-MH-S-DK protocol	1 (20%)	0	4 (80%)
Transfer protocols	3 (60%)	1 (20%)	1 (20%)
Safety protocols	3 (60%)	0	2 (40%)
Quality improvement protocols	0	2 (40%)	3 (60%)
Interventions			
Vital signs	4 (80%)	1 (20%)	0
Airway	5 (100%)	0	0
Breathing	4 (80%)	1 (20%)	0
Volume resuscitation	5 (100%)	0	0
Procedures and safe blood transfusions	4 (80%)	1 (20%)	0
Cardiac intervention	4 (80%)	1 (20%)	0
Seizures and unconsciousness	3 (60%)	2 (40%)	0
Sepsis control	3 (60%)	1 (20%)	1 (20%)
Trauma care	3 (60%)	2 (40%)	0
Vaginal delivery, uterotonic medication and neonatal resuscitation	3 (60%)	0	2 (40%)
Essential equipment	3 (60%)	1 (20%)	1 (20%)

## Data Availability

Data sharing not applicable to this article as no datasets were generated or analysed during the current study.
